# Blurring Fact and Fiction: A Complex Case of Recurrent Emergency Presentations, Polypharmacy, and Psychiatric Conflict

**DOI:** 10.7759/cureus.98979

**Published:** 2025-12-11

**Authors:** Bola Reyad

**Affiliations:** 1 General Internal Medicine, St. Mary's Hospital, Isle of Wight NHS Trust, Isle of Wight, GBR

**Keywords:** anorexia nervosa, factitious disorder, hospital dependency, medication misuse, non-epileptic attack disorder, personality disorder, polypharmacy, psychosomatic illness, self-induced hypoglycaemia, semaglutide misuse

## Abstract

A patient in their early 20s with a complex psychiatric and medical history presented with severe electrolyte imbalance, dehydration, and progressive functional decline. After initial stabilisation, the patient developed recurrent episodes of hypotension, hypoglycaemia, and seizure-like activity despite normal neuroimaging and an established diagnosis of non-epileptic attack disorder. Repeated nursing safety checks revealed concealed medications, including antihypertensives, anticoagulants, semaglutide, and central nervous system depressants, all temporally associated with clinical deterioration and emergency responses. The patient’s presentation raised concern for factitious disorder, compounded by comorbid anorexia nervosa, emotionally unstable personality disorder, and complex PTSD.

This case highlights the diagnostic and ethical challenges of managing medically unstable patients whose presentations blur the boundaries between psychiatric and somatic illness. It illustrates the risks of reinforcing maladaptive behaviour through repeated interventions and prolonged hospitalisation, emphasising the importance of multidisciplinary collaboration, cautious escalation of care, and early involvement of psychological and ethical support.

## Introduction

A patient in their early 20s with a complex psychiatric and medical background, including anorexia nervosa, emotionally unstable personality disorder, complex PTSD, ADHD, and non-epileptic attack disorder (NEAD), presented with severe electrolyte imbalance, dehydration, and functional decline. After initial stabilisation, they developed recurrent hypotension, hypoglycaemia, and seizure-like activity despite normal neuroimaging. During routine safety checks, suspected concealed access to non-prescribed medications (antihypertensives, diuretics, glucagon-like peptide-1 (GLP-1) agonists, and sedatives) was identified in close temporal association with several deteriorations. The patient had previously worked as a healthcare assistant and engaged extensively with medical information, complicating assessment and risk management. Nasogastric (NG) feeding was used but repeatedly interrupted by self-removal; escalation to nasojejunal (NJ) feeding was discussed and declined at multidisciplinary team (MDT) review, as the anticipated harm outweighed the benefit. Hospital pharmacy leadership and safeguarding teams initiated a formal review into medication-access pathways.

This case underscores the diagnostic and ethical challenges at the interface of psychiatric and medical illness. It highlights the importance of consistent multidisciplinary planning, clear therapeutic boundaries, and early involvement of pharmacy and safeguarding.

## Case presentation

The patient, with a history of anorexia nervosa, emotionally unstable personality disorder, complex PTSD, ADHD, and NEAD, was admitted in late 2024 with nausea, dehydration, and significant weight loss (body mass index 13.5 kg/m²). They had a long-standing pattern of hospital dependence, self-harm, and recurrent admissions across both medical and psychiatric services. The patient had previously worked as a healthcare assistant and demonstrated a strong interest in medical knowledge, frequently reading about medications and clinical conditions, which complicated clinical assessment and boundary setting.

Initial investigations revealed marked electrolyte disturbances, including hypokalaemia (2.6 mmol/L), hypophosphataemia, and hypomagnesaemia. Intravenous fluids, thiamine, and electrolyte replacement were commenced, followed by cautious refeeding. During admission, the patient developed a *Staphylococcus aureus* peripherally inserted central catheter infection, complicated by an ipsilateral deep-vein thrombosis.

After initial stabilisation, the patient experienced recurrent episodes of profound hypotension (systolic blood pressure 50-60 mmHg) and symptomatic hypoglycaemia (1.8-3 mmol/L), often accompanied by seizure-like activity. Prior electroencephalograms supported a diagnosis of NEAD rather than epilepsy. These episodes triggered repeated emergency responses and brief transfers to the high-dependency unit.

During one such deterioration, the patient was found drowsy with an odour of alcohol noted. Tablets of moxonidine (400 µg) and furosemide (40 mg) were discovered nearby. Subsequent routine nursing safety checks identified suspected concealed access to antihypertensives, diuretics, glucose-lowering agents, sedatives, and GLP-1 receptor agonists. Several episodes of clinical deterioration occurred shortly after suspected ingestion of these agents (Table 1). The patient declined toxicology testing and denied ingestion. A psychiatric review was obtained, and a temporary Section 5(2) order under the Mental Health Act was applied for a safety assessment, which was rescinded following reassessment.

**Table 1:** Summary of suspected concealed medication classes identified during routine ward safety checks Medication classes suspected to have been accessed or concealed by the patient. These agents may have contributed to physiological instability through hypotension, hypoglycaemia, or reduced consciousness. GLP-1: glucagon-like peptide-1, CNS: central nervous system

Medication class	Examples	Main clinical effect/risk
Antihypertensives	Moxonidine	Hypotension; bradycardia
Diuretics	Furosemide	Volume depletion; electrolyte loss
Hypoglycaemic agents	GLP-1 receptor agonists; oral glucose-lowering drugs	Hypoglycaemia
Sedatives/others	Various CNS-active agents	Drowsiness; central nervous system depression

Feeding and nutrition were managed via NG feeding, which was repeatedly interrupted by self-removal, sometimes attributed to accidental traction by a friend. The patient persistently requested NJ feeding, but following several MDT meetings involving gastroenterology, radiology, general medicine, dietetics, and psychiatry, it was agreed that NJ feeding would likely reinforce maladaptive behaviour and pose more harm than benefit, so this intervention was not pursued.

**Figure 1:**
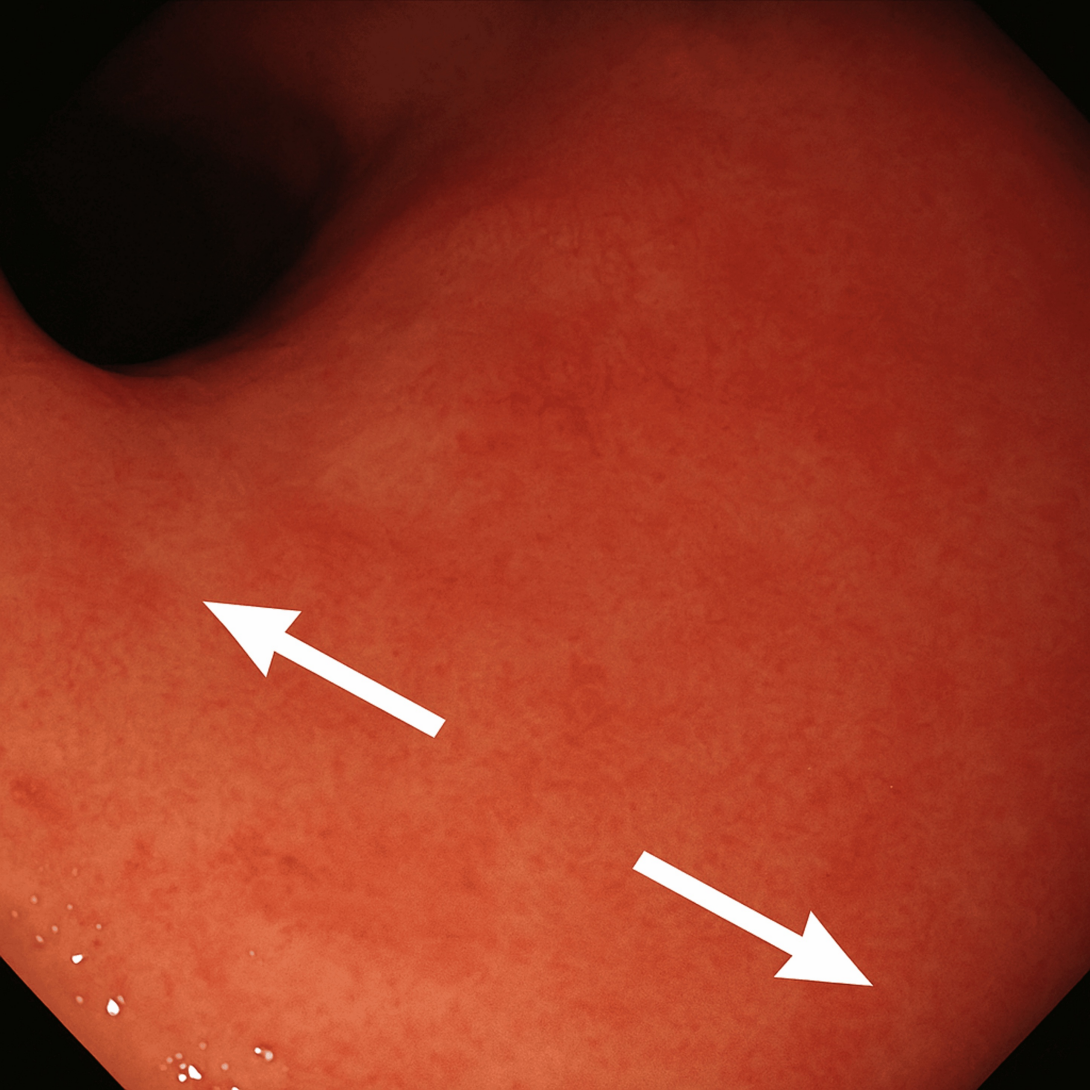
Normal gastric antral mucosa on upper gastrointestinal endoscopy Endoscopy performed in April 2025 shows normal antral mucosa (arrows) without ulceration, bleeding, or structural abnormality. The oesophagus and duodenum were also unremarkable.

Further investigations included normal brain magnetic resonance imaging and a normal transthoracic echocardiogram. Oesophagogastroduodenoscopy demonstrated normal gastric, oesophageal, and duodenal mucosa without ulceration or obstruction (Figure 1). Gastric-emptying studies confirmed delayed gastric emptying. Representative biochemical results across multiple admissions are presented in Table 2, demonstrating preserved renal function and low inflammatory activity despite repeated episodes of hypotension and hypoglycaemia.

**Table 2:** Representative biochemical parameters recorded across multiple admissions (March-June 2025) Results demonstrate preserved renal function and low inflammatory activity despite recurrent reported hypoglycaemia, hypotension, and collapse. CRP: C-reactive protein

Date	Sodium (mmol/L)	Potassium (mmol/L)	Urea (mmol/L)	Creatinine (µmol/L)	CRP (mg/L)
26-Mar-25	139	3.5	–	–	0.8
16-Apr-25	142	3.3	–	–	0.5
04-May-25	142	3.9	–	–	1.8
16-May-25	136	3.3	–	–	38.5
19-May-25	140	3.6	3.7	65	4.7
22-May-25	141	3	1.5	69	1
09-Jun-25	136	3.4	3.7	65	0.2
12-Jun-25	142	3.1	2.6	67	0.2

Because of the repeated findings of suspected non-prescribed medication use, the issue was escalated to hospital pharmacy leadership and safeguarding teams. Safety managers initiated a formal review and began tracing possible supply routes. Information available suggested the patient might have obtained medications through a friend using a non-hospital address, but the exact source remained unclear. The matter was referred to the appropriate governance authorities for continued investigation.

At the time of reporting, the patient remained under joint medical and psychiatric follow-up with a unified management plan centred on nutritional rehabilitation, electrolyte replacement, psychological therapy, and ongoing safeguarding oversight to minimise risk and unnecessary medical interventions.

## Discussion

Diagnostic complexity

Patients with overlapping psychiatric and medical conditions present significant diagnostic challenges, particularly when symptoms fluctuate and investigations provide limited clarity. In this case, recurrent episodes of hypoglycaemia, hypotension, and collapse created uncertainty regarding their underlying cause. Although initial presentations appeared physiological, the repeated and temporally associated discovery of suspected non-prescribed medications, including moxonidine, furosemide, and GLP-1 receptor agonists, raised concern for self-induced clinical deterioration [1,2].

Factitious disorder involves the intentional production or exaggeration of symptoms without external reward and is usually driven by complex, often unconscious psychological mechanisms [3]. Differentiating factitious behaviour from genuine illness or functional presentations is especially difficult in individuals with long-standing psychiatric comorbidities such as eating disorders, personality disorders, and PTSD, particularly when they possess above-average medical knowledge or prior healthcare experience. In this case, the patient’s previous work as a healthcare assistant and extensive online engagement with medical information further complicated assessment and risk management.

Ethical, governance, and multidisciplinary considerations

This case also highlights the ethical and governance dilemmas that arise when patient autonomy intersects with clinical safety. The temporary use of Section 5(2) powers under the Mental Health Act served as a short-term measure to allow assessment when covert ingestion was suspected. Ethical discussions centred on balancing respect for autonomy with the duty to prevent harm. Early involvement of psychiatry, the hospital ethics team, and pharmacy governance provided a structured framework for decision-making.

Because of the repeated discovery of suspected concealed medications, the case was escalated to hospital pharmacy leadership and safeguarding teams. A formal review led by safety managers was initiated to trace potential sources and implement systems-level controls on medication access. Information suggested that the patient may have obtained drugs through a friend using a non-hospital address; the matter was handed over to governance authorities for further investigation.

Multidisciplinary cooperation remained central. Collaboration among gastroenterology, psychiatry, endocrinology, acute medicine, and nursing produced a unified plan. The collective decision to avoid nasojejunal feeding, an intervention that risked reinforcing maladaptive behaviour, reflected a careful balance between physiological need and psychological risk. Objective investigations, including endoscopy (Figure 1) and serial biochemical monitoring (Tables 1-2), helped exclude organic disease and redirected focus toward behavioural and psychological rehabilitation.

Practical safeguards (implications for practice)

Practical safeguards include early escalation to pharmacy governance and safeguarding teams whenever clinical deterioration appears disproportionate to objective findings or linked to non-prescribed agents. Ward-level protocols for belongings checks and medication security should be clearly defined and aligned with institutional policies. Nutrition strategies must avoid reinforcing dependence on invasive, declining-benefit NJ feeding when the behavioural risk outweighs the potential benefit. Finally, unified documentation and communication, such as MDT summaries and electronic alerts, are essential to maintain consistency across teams and reduce staff distress.

## Conclusions

This case highlights the complexity of managing patients who present with both genuine medical illness and behaviours suggestive of factitious disorder. Recurrent collapses, suspected access to non-prescribed medications, and fluctuating engagement created diagnostic uncertainty and ethical tension that demanded coordinated, multidisciplinary input. A structured team approach, incorporating medical, psychiatric, and governance perspectives, proved essential in maintaining safety and preventing reinforcement of maladaptive behaviours. Early involvement of pharmacy governance and safeguarding teams provided system-level oversight and ensured that medication access concerns were addressed appropriately.

Overall, this case underlines the importance of integrating psychological insight with medical reasoning, maintaining clear communication and firm yet compassionate boundaries, and promoting continuity of care within a consistent multidisciplinary framework to achieve stability and rebuild therapeutic trust.
